# The DREAM complex promotes gene body H2A.Z for target repression

**DOI:** 10.1101/gad.255810.114

**Published:** 2015-03-01

**Authors:** Isabel Latorre, Michael A. Chesney, Jacob M. Garrigues, Przemyslaw Stempor, Alex Appert, Mirko Francesconi, Susan Strome, Julie Ahringer

**Affiliations:** 1 The Gurdon Institute, University of Cambridge, Cambridge CB2 1QN, United Kingdom;; 2 Molecular, Cell, and Developmental Biology, University of California at Santa Cruz, Santa Cruz, California 95064, USA;; 3 EMBL-CRG Systems Biology Unit, Centre for Genomic Regulation (CRG), Universitat Pompeu Fabra (UPF), Barcelona 08003, Spain

**Keywords:** *C. elegans*, H2A.Z, Retinoblastoma/DREAM, transcriptional repression

## Abstract

The DREAM (DP, Retinoblastoma [Rb]-like, E2F, and MuvB) complex controls cellular quiescence by repressing cell cycle genes, but its mechanism of action is poorly understood. Here we show that *Caenorhabditis elegans* DREAM targets have an unusual pattern of high gene body HTZ-1/H2A.Z. In mutants of *lin-35*, the sole p130/Rb-like gene in *C. elegans*, DREAM targets have reduced gene body HTZ-1/H2A.Z and increased expression. Consistent with a repressive role for gene body H2A.Z, many DREAM targets are up-regulated in *htz-1*/H2A.Z mutants. Our results indicate that the DREAM complex facilitates high gene body HTZ-1/H2A.Z, which plays a role in target gene repression.

## Abstract (precis)

The DREAM (DP, Retinoblastoma [Rb]-like, E2F, and MuvB) complex controls cellular quiescence by repressing cell cycle genes. Latorre et al. show that *Caenorhabditis elegans* DREAM targets have an unusual pattern of high gene body HTZ-1/H2A.Z. In mutants of *lin-35*, the sole p130/Rb-like gene in *C. elegans*, DREAM targets have reduced gene body HTZ-1/H2A.Z and increased expression. Consistent with a repressive role for gene body H2A.Z, many DREAM targets are up-regulated in *htz-1*/H2A.Z mutants.

The DREAM (DP, Retinoblastoma [Rb]-like, E2F, and MuvB) complex is a key regulator of cellular quiescence, and its eight core components are conserved in worms, flies, and humans (Korenjak et al. 2004; Lewis et al. 2004; Harrison et al. 2006; Litovchick et al. 2007). In mammals, DREAM represses cell cycle progression genes (Litovchick et al. 2007; for review, see Sadasivam and DeCaprio 2013). Many of the same genes are also targets of the homologous complex in worms and flies—DRM in *Caenorhabditis elegans* and dREAM in *Drosophila*—where the complex inhibits cell cycle progression and also regulates genes involved in differentiation and germline development (Sadasivam and DeCaprio 2013). As dysregulation of DREAM components and its targets is frequent in cancer, understanding its mechanism of action is an important area of study.

DREAM consists of an Rb-like pocket protein, p130; an E2F protein and its heterodimeric DP partner; and a five-component MuvB core (LIN9, LIN37, LIN52, LIN54, and RBBP4). Although the conserved requirement for DREAM in repression of cell cycle genes is well documented, DREAM does not possess any known enzymatic activity, and how the complex mediates repression is poorly understood. Components of DREAM can interact with a variety of other chromatin regulators, such as the Sin3B repressor complex, histone deacetylases (HDACs), Swi/Snf components, and Polycomb-repressive complexes, implicating interacting factors as contributing to repression (Blais and Dynlacht 2007; Grandinetti and David 2008; Dimova 2011; Fiorentino et al. 2013). Here we show that enrichment of the histone variant H2A.Z/HTZ-1 on target gene bodies is part of the repression mechanism of DREAM in *C. elegans.*

## Results and Discussion

### Genome-wide profiling of DREAM binding

**Figure 1.**
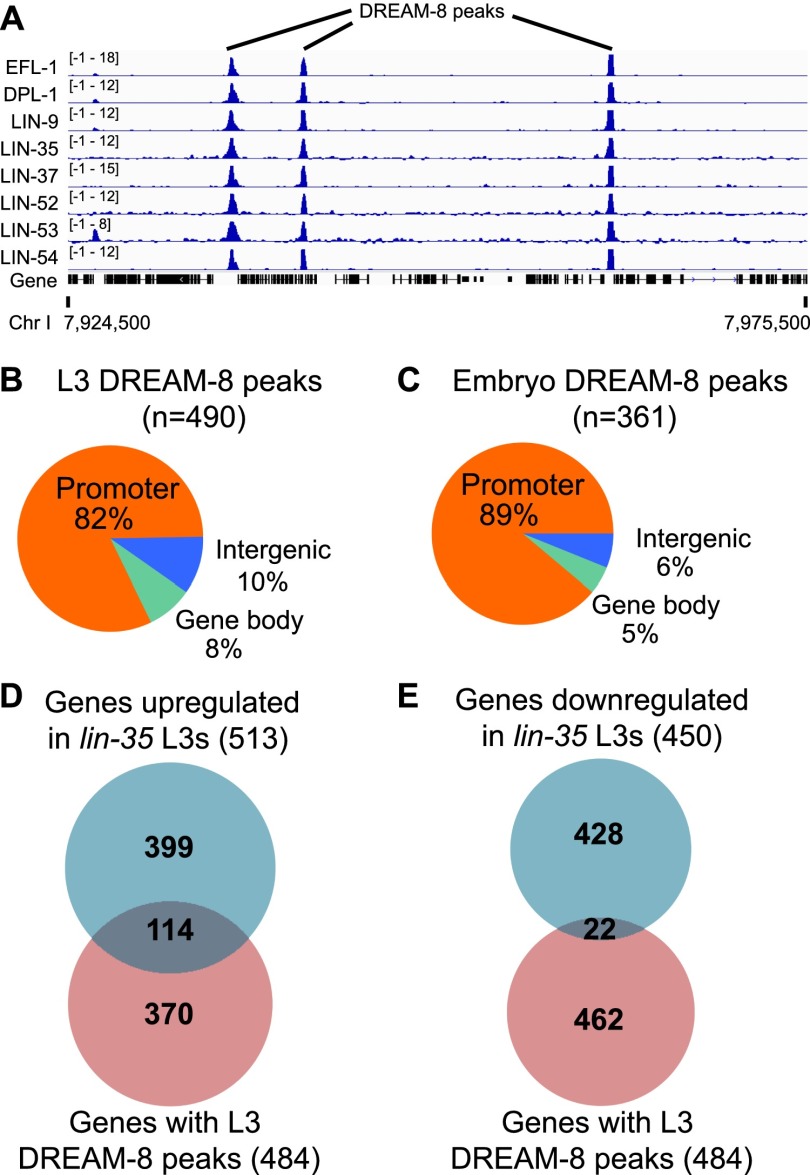
Features of *C. elegans* DREAM peaks. (*A*) Genome browser view of a region from ChrI showing *Z*-score normalized ChIP-seq signal for all eight DREAM complex subunits at the L3 stage. (*B*,*C*) Locations of L3 stage (*B*) and embryo (*C*) DREAM-8 peak regions with respect to genes. (*D*,*E*) Venn diagrams of the overlap between L3 stage DREAM-8 genes and genes up-regulated (*D*) or down-regulated (*E*) in *lin-35(n745)* mutant L3s.

We profiled the genomic distribution of the eight *C. elegans* DREAM complex members (LIN-35/Rb-like p130, EFL-1/E2F, DPL-1/DP1, LIN-9, LIN-37, LIN-52, LIN-54, and LIN-53/RBBP4) in L3 larvae and embryos using chromatin immunoprecipitation (ChIP) followed by high-throughput sequencing (ChIP-seq) (Fig. 1A; see the Materials and Methods; Supplemental Table S1). The data sets overlap substantially with previously published binding data for individual DREAM proteins at various stages and in various tissues (LIN-54, LIN-35, EFL-1 and DPL-1) (Supplemental Fig. S1; Tabuchi et al. 2011; Kudron et al. 2013). DREAM complex members bound 1619 regions in L3 larvae and 2076 in embryos. Of these, 1350 regions overlapped, indicating similar targets at the two stages (Supplemental Fig. S2A; Supplemental Table S2). Differences in the numbers of peaks identified per factor and per stage appear to be primarily technical; we did not see enrichment for particular subgroupings of factors (see the Materials and Methods; data not shown). Overall, we defined 490 high-confidence sites bound by all eight DREAM members (DREAM-8) in L3 and 361 in embryos (Supplemental Table S1; Supplemental Fig. S2B). These sites showed significant enrichment for motifs previously associated with the DREAM components EFL-1 and LIN-54 (Supplemental Fig. S3; Kirienko and Fay 2007; Tabuchi et al. 2011; Kudron et al. 2013). In this study, we focus on the high confidence DREAM-8 binding sites.

### DREAM is associated with broadly expressed genes

**Figure 2.**
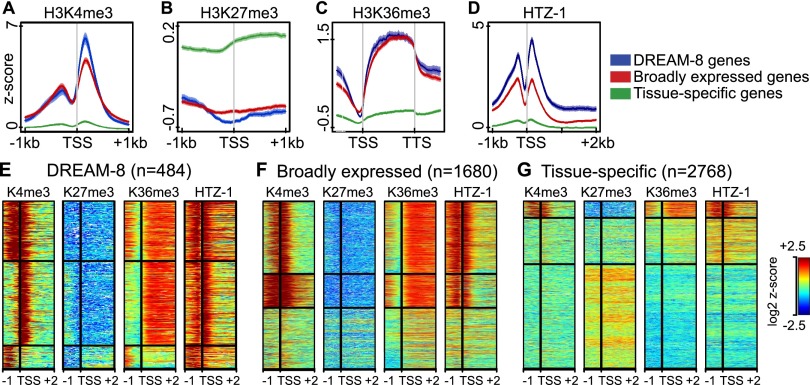
DREAM-bound genes display features of broadly expressed genes. (*A–D*) Average *Z*-score profiles for the specified gene sets of normalized signal/input of H3K4me3 (*A*) and H3K27me3 ± 1 kb centered on the transcription start site (TSS) (*B*), H3K36me3 ± 1-kb region from anchors on the TSS and transcription termination site (TTS) (*C*), and HTZ-1 1 kb upstream of and 2 kb downstream from the TSS (*D*). (*E–G*) Heat map analysis of log_2_ *Z*-scores of normalized signal/input for the indicated features and gene sets over regions extending from 1 kb upstream of the TSS to 2 kb downstream. To aid visualization of patterns, genes were separated into three groups using K-means clustering.

We found that most DREAM-8 sites are located at promoters (Fig. 1B,C; Supplemental Table S3). At both the embryo and L3 stages, DREAM-8 genes have intermediate to high levels of gene expression (Supplemental Fig. S4A,B). Markers of gene activity (H3K4me3, H3K36me3, and HTZ-1/H2A.Z) and repression (H3K27me3) on DREAM-8 genes have patterns similar to those on broadly expressed genes and dissimilar to profiles on tissue-specific genes, suggesting that DREAM-8 genes are likely to be broadly expressed (Fig. 2A–G). We also observed that many DREAM-8 genes have an unusually high level of HTZ-1/H2A.Z on their transcribed regions (Fig. 2E), which we investigate below.

### Identification of direct DREAM target genes

To investigate gene expression regulation by DREAM, we carried out gene expression profiling by RNA sequencing (RNA-seq) at the L3 stage, identifying 963 misregulated genes in *lin-35/*Rb-like p130 mutants (Supplemental Table S4). Of the 484 genes bound by DREAM-8 at the L3 stage, 28% (136 genes) were misexpressed in *lin-35* mutants, with 84% of these up-regulated, consistent with previous studies indicating that DREAM generally functions as a transcriptional repressor (Fig. 1D,E; Chi and Reinke 2006; Kirienko and Fay 2007; Petrella et al. 2011). Similar to DREAM targets in humans, the DREAM-8 genes up-regulated in *lin-35* mutants are highly enriched for cell cycle and cell division-related functions (Supplemental Table S5; Litovchick et al. 2007). Using *lin-35* expression data as a proxy for DREAM function, we define direct DREAM targets as genes up-regulated in *lin-35* mutants and bound by DREAM-8 and indirect DREAM targets as those up-regulated in *lin-35* mutants but not bound by any DREAM member.

### Direct DREAM targets are repressed but not completely silenced

**Figure 3.**
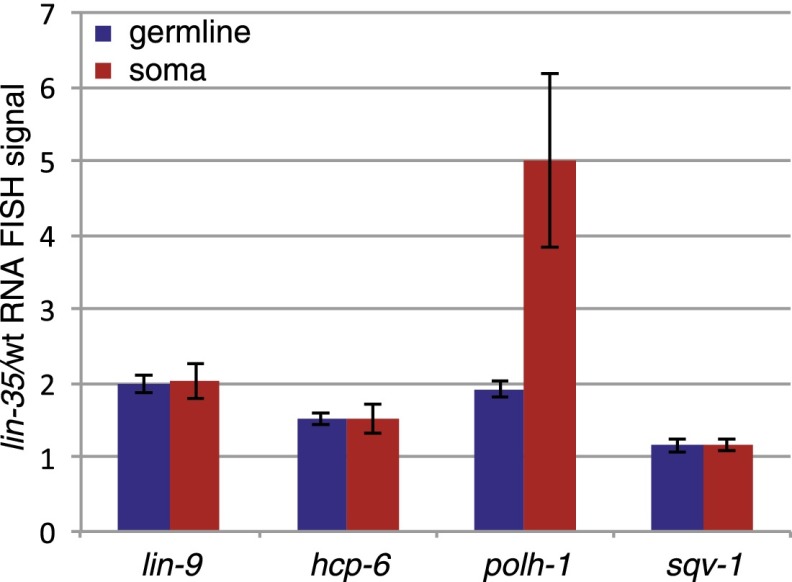
Direct DREAM target genes are up-regulated in *lin-35* mutants. Bar chart of RNA-FISH signal ratio in *lin-35* mutants compared with wild-type L3 larvae (ratio of number of fluorescent foci) using probes directed against direct DREAM targets *lin-9*, *hcp-6*, and *polh-1* and a control gene, *sqv-1*. RNA-FISH signal was analyzed separately in soma and germline. Error bars show standard errors. Representative images are shown in Supplemental Figure S5.

Increased expression of target genes at the L3 stage could be due to increased expression in cells that already express the gene or ectopic expression in cells that normally do not express it. To distinguish between these possibilities, we used RNA-FISH to analyze the gene expression pattern of three DREAM targets (*hcp-6*, *lin-9*, and *polh-1*) and a nonregulated control gene (*sqv-1*) in wild-type and *lin-35* mutant animals. We found that all four tested genes showed germline and broad somatic expression in wild-type animals (Fig. 3; Supplemental Fig. S5A). In *lin-35* mutants, the three DREAM targets exhibited increased RNA-FISH signal in their normal sites of expression in the germline and soma (Fig. 3; Supplemental Fig. S5B). In contrast, the control gene *sqv-1* showed no change in RNA-FISH signal (Fig. 3). These results suggest that the DREAM complex acts to reduce the expression of its targets rather than completely silence expression.

### DREAM facilitates gene body enrichment of HTZ-1/H2A.Z on its direct targets

**Figure 4.**
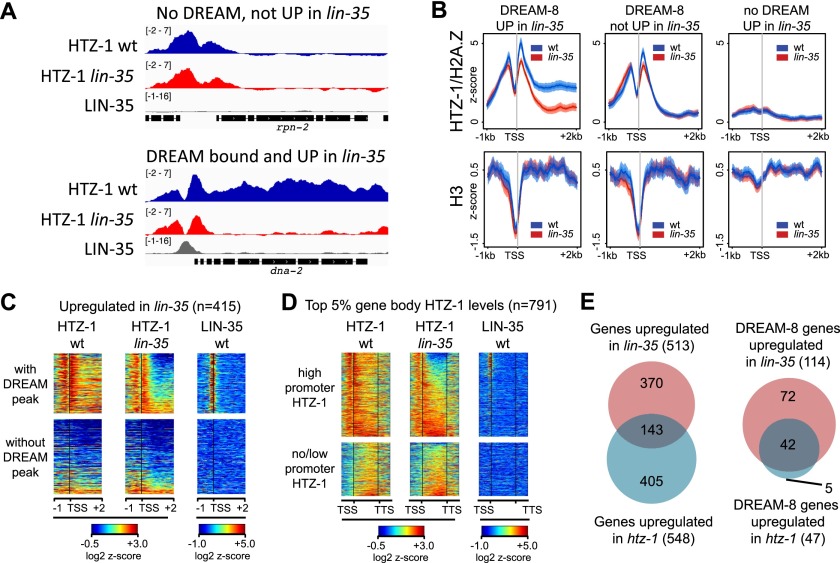
HTZ-1 is enriched on the gene bodies of direct DREAM targets. (*A*) Genome browser views of HTZ-1 in wild type and *lin-35* mutants and LIN-35 in wild-type L3 stage larvae of a gene neither bound nor regulated by DREAM (*top*) and a direct DREAM target (*bottom*); signals are *Z*-score profiles of normalized signal/input. (*B*) Average *Z*-score profiles of normalized signal/input for the indicated gene sets and features at the L3 stage ([blue] wild type; [red] *lin-35* mutant), extending from 1 kb upstream of to 2 kb downstream from the TSS. (*C*) Heat map analysis of log_2_ *Z*-score normalized signal/input for HTZ-1 in wild type and *lin-35* mutants and LIN-35 in wild type, plotted on *lin-35* up-regulated genes (*n* = 415). Genes were sorted into those with and without DREAM peaks in L3 and by gene body HTZ-1 level in *lin-35* mutants. (*D*) Heat map analysis of log_2_ *Z*-score normalized signal/input for genes in the top 5% in gene body HTZ-1 levels (*n* = 791). Genes were separated into two clusters based on promoter HTZ-1 level in the region from the TSS − 500 base pairs (bp) to the TSS + 200 bp and then sorted by gene body HTZ-1 levels in *lin-35* mutants. (*E*) Venn diagrams of the overlap between genes up-regulated in *lin-35* and *htz-1* mutant L3 larvae. (*Left*) All genes up-regulated in *lin-35* versus *htz-1*. (*Right*) DREAM-8 genes up-regulated in *lin-35* versus *htz-1*.

The high levels of HTZ-1/H2A.Z on the transcribed regions of some DREAM-bound genes (Fig. 2D,E) prompted us to investigate the relationship between DREAM binding and HTZ-1/H2A.Z pattern. Broadly expressed genes and DREAM-8 genes typically exhibit sharp localization of HTZ-1/H2A.Z at their promoters (Fig. 4A,B). Notably, many direct DREAM targets additionally show HTZ-1/H2A.Z enrichment along the gene body (Fig. 4A–C). By comparison, most genes up-regulated in *lin-35* mutants but not bound by DREAM (indirect targets) do not exhibit HTZ-1/H2A.Z enrichment on either the promoter or gene body (Fig. 4B,C).

To further explore the relationship between DREAM and HTZ-1/H2A.Z, we determined whether a high level of gene body HTZ-1/H2A.Z (top 5%) was associated with direct DREAM regulation. Indeed, 75% of direct DREAM-8 targets are in the top 5% of genes with high levels of gene body HTZ-1 (*P* = 6.40 × 10^−82^) (Supplemental Table S6), and these comprise 10% of high HTZ-1 gene body genes. As the DREAM-8 class is a small, high-confidence set of genes bound by all eight DREAM subunits, we also looked at the association of high gene body HTZ-1 with genes bound by at least one DREAM subunit (*n* = 1087). We found that 22% of these genes have high gene body HTZ-1, and these comprise 30% of the high HTZ-1 gene body group (*P* = 1.14 × 10^−95^) (Supplemental Table S6). Furthermore, among genes bound by any DREAM subunit that are also up-regulated in *lin-35* (*n* = 185), 71% have high gene body HTZ-1 (*P* = 4.28 × 10^−131^) (Supplemental Table S6). In contrast, genes bound by DREAM-8 but not misregulated in *lin-35* mutants are not significantly enriched for having high gene body HTZ-1 levels (*P* = 0.06). We also observed that LIN-35 is primarily associated with genes that have high levels of HTZ-1 at both promoters and gene bodies (Fig. 4D). These results show that high gene body HTZ-1/H2A.Z is strongly associated with DREAM-mediated transcriptional repression.

To ask whether gene body HTZ-1/H2A.Z is relevant to DREAM function, we investigated its genome-wide distribution in *lin-35* mutants. This revealed a striking and specific loss of gene body HTZ-1 on direct DREAM targets, with promoter enrichment remaining high (Fig. 4B). The loss of HTZ-1 on gene bodies is not due to a general reduction of nucleosome abundance, as histone H3 levels on DREAM targets were not changed in *lin-35* mutants (Fig. 4B). High HTZ-1 in DREAM target gene bodies is also not a consequence of general spreading of markers of active promoters into gene bodies, as two other such markers of active promoters, H3K4me3 and H4ac4, are enriched only at promoters and not on the gene bodies of DREAM targets. In *lin-35* mutants, levels of these latter modifications were increased at DREAM target gene promoters, consistent with increased expression (Supplemental Fig. S6). We conclude that DREAM facilitates enrichment of HTZ-1/H2A.Z on the gene bodies of its targets and that this pattern is associated with target gene repression. Gene body enrichment of H2A.Z could be through promoting its deposition or preventing its removal, either directly or indirectly.

### HTZ-1/H2A.Z regulates DREAM target genes

If HTZ-1/H2A.Z is functionally linked to DREAM and enrichment of HTZ-1/H2A.Z on the gene bodies of DREAM targets contributes to their repression, then loss of HTZ-1/H2A.Z activity would be expected to cause gene expression changes similar to those caused by loss of LIN-35. To test this, we analyzed gene expression in homozygous *htz-1*-null mutants produced by heterozygous mothers. Loss of maternal *htz-1* via RNAi causes embryonic lethality, but due to the maternal load of wild-type gene product, *htz-1* homozygotes from heterozygous mothers develop into sterile adults, allowing assessment of a partial loss of HTZ-1 function in larvae (Updike and Mango 2006; Whittle et al. 2008).

We observed a large overlap in gene expression changes in *lin-35* and *htz-1* mutants at the L3 stage. Of the 1245 genes misexpressed in *htz-1* mutants, 28% were also misexpressed in *lin-35* mutants (*P* = 1 × 10^−149^), with 97% of the changes concordant in the direction of change (up or down) (Supplemental Table S4; Fig. 4E). Consistent with a repressive role for gene body enrichment of HTZ-1, most (83%) genes in the top 5% of gene body HTZ-1 levels that were misexpressed in *htz-1* were up-regulated (Supplemental Table S6). Furthermore, most (89%) DREAM-bound genes that were up-regulated in *htz-1* were also up-regulated in *lin-35* (Fig. 4E). We conclude that HTZ-1 and LIN-35 function in a common pathway to repress DREAM target genes.

### Conclusions

H2A.Z has multiple regulatory roles and has been associated with both transcriptional activity and repression. This histone variant is most commonly observed at gene promoters, where it is positively correlated with gene expression in higher eukaryotes (Barski et al. 2007; Mavrich et al. 2008; Whittle et al. 2008; Hardy et al. 2009; Jin et al. 2009). Indeed, HTZ-1/H2A.Z is enriched at the promoters of many broadly expressed genes in *C. elegans*, and this enrichment is not strongly affected in *lin-35* mutants.

In contrast, gene body deposition of H2A.Z has been associated with reduced transcription in diverse systems, including mammals, plants, and yeast (Barski et al. 2007; Hardy et al. 2009; Coleman-Derr and Zilberman 2012a). Some studies support an association between H2A.Z distribution on gene bodies and gene expression only under certain environmental conditions, such as in response to temperature changes or environmental stress (Coleman-Derr and Zilberman 2012b). In this study, we found that H2A.Z is highly enriched on the gene bodies of direct DREAM target genes, many of which are involved in cell cycle progression. Like environment-responsive genes, expression of cell cycle genes is highly regulated.

Although a mechanistic link between H2A.Z and DREAM has not been previously reported, a model of cooperative gene repression is supported by several previous studies. In *Drosophila*, reduction of function of H2A.Z or four different DREAM components similarly suppressed a cytokinesis defect (DeBruhl et al. 2013). A *Drosophila* E2F target gene reporter is also derepressed by loss of components of the Swr1 complex, which is involved in H2A.Z deposition (Lu et al. 2007). Furthermore, mutants of both H2A.Z and Domino, which encodes a Swr1 complex protein, suppress the eye defects of a hypomorphic allele of cyclin E, an E2F target that is also suppressed by the DREAM component Rbf1 (Lu et al. 2007). In addition, mutants of several *C. elegans* orthologs of the Tip60/NuA4 complex, which facilitates H2A.Z deposition, genetically interact with or share mutant phenotypes with mutants of DREAM member genes (Ceol and Horvitz 2004; Poulin et al. 2005; Andersen et al. 2008). Given the conservation of DREAM components and targets across species, it is likely that the relationship between H2A.Z and DREAM that we uncovered in *C. elegans* applies more generally. Our results provide a new insight into the mechanism of DREAM function and suggest a new avenue for the study of Retinoblastoma-related proteins.

## Materials and methods

### Worm culture and strains

The following strains were used and cultured using standard methods: *N2*, *lin-35(n745)* outcrossed 5× (kindly provided by D. Fay), and VC2480 [*htz-1(ok3099) IV/nT1(qIs51) IV;V*].

### ChIP-seq and RNA-seq experiments

Antibodies used for ChIP are described in Supplemental Table S8. Collection of wild-type or *lin-35* mutant L3 larvae, extract preparation, and ChIP were performed as in Kolasinska-Zwierz et al. (2009) with the following modifications. Cross-linked chromatin was sonicated using a Bioruptor (Diagenode) to an average size of 250 baase pairs (bp). After ChIP and DNA purification, libraries were prepared using the Illumina TruSeq kit. Fragments in the 250–350 bp range were size-selected by gel extraction and sequenced on the Illumina platform. Embryo extracts and ChIPs were performed as in Rechtsteiner et al. (2010), except that late stage embryos (aged 3.5 h after being obtained from gravid hermaphrodites) were collected and frozen prior to fixation; extracts were prepared as described above for L3 larvae; and the Illumina ChIP-seq DNA sample preparation kit was used for library preparation.

*N2*, *lin-35*, and *htz-1* larvae for RNA-seq were grown at 20°C and harvested at the L3 stage. *htz-1* homozygotes were isolated from a mixed population that also contained *htz-1/nT1[qIs51]* larvae by using a COPAS worm sorter (Union Biometrica). Total RNA was extracted using TriPure (Roche) and further purified using an RNeasy column (Qiagen). RNA-seq libraries were prepared from 2 µg of total RNA using the Illumina TruSeq RNA kit according to the manufacturers’ instructions and sequenced on the Illumina platform.

### Data processing and plotting

In short, ChIP-seq and RNA-seq reads were aligned to the WS220/ce10 assembly of the *C. elegans* genome using BWA version 0.6.2 (Li and Durbin 2009) with default settings (BWA-backtrack algorithm). ChIP-seq data were normalized using the BEADS algorithm implemented in R (Cheung et al. 2011). Where available, we used transcription start sites (TSSs) recently identified based on capped RNA-seq (Chen et al. 2013; Kruesi et al. 2013). If none were available, we used WormBase annotated TSSs. Average signal plots and heat maps for histone marks and DNA-associated factors were created using in-house tools. Venn diagrams were generated using the R package VennDiagram (Chen and Boutros 2011). Statistical analyses of Venn overlapping regions were performed using hypergeometric probability functions in R. Motif analyses were performed on L3 DREAM-8 peak overlap regions using the Seqpos motif tool in the Cistrome toolbox with default parameters (Liu et al. 2011). Peak calling for ChIP-seq tracks was done using the wignorm utility from MACS14 (Zhang et al. 2008). Differential gene expression between N2, the *lin-35* mutant, and the *htz-1* mutant was tested using DESeq2 (Love et al. 2014). Supplemental Table S5 gives log_2_ fold change (FC), statistical significance estimates, and reads per kilobase per million mapped reads (RPKM) values for each gene. The average HTZ-1 ChIP-seq signal on gene body regions (from 500 bp downstream from the WormBase gene start to the gene end, excluding genes <500 bp in length) was extracted from BEADS normalized tracks using the bigWigSummary utility from University of California at Santa Cruz user tools (Kent et al. 2010). Mean signals, log_2_ FC between wild-type and mutant samples, and the statistical significance estimate (*P*-value) of this change are in Supplemental Table S6. Full details of data processing are in the Supplemental Material.

### RNA-FISH

*N2* and *lin-35* larvae were fixed and stained by RNA-FISH as described (Raj et al. 2008). Stellaris FISH probes targeting *hcp-6*, *lin-9*, *polh-1*, and *sqv-1* were obtained from Bioresearch Technologies. Samples were imaged on an Axioplan 2 microscope fitted with an LSM 510 confocal detection system (Carl Zeiss). For each animal, FISH signals in the germline and soma were counted in three or four focal planes across the gonad; the position of the germline was determined from DAPI staining of nuclei. Images from two or three animals (seven to 12 focal planes) were used per experiment. For each probe, average counts in germline or soma of *lin-35(n745)* were divided by average counts in wild type to determine relative mRNA expression.

### Data access

The ChIP-seq and RNA-seq data used in this study are available from Gene Expression Omnibus (GEO; http://www.ncbi.nlm.nih.gov/geo) or modENCODE (http://intermine.modencode.org) (Supplemental Table S9).

## Supplementary Material

Supplemental Material
