# Supplementary material for: The DREAM complex promotes gene body H2A.Z for target repression
Source: Genes Dev. 2015 Mar 1;29(5):495–500. doi: 10.1101/gad.255810.114 (PMC4358402; doi:10.1101/gad.255810.114)
Supplement: Supplemental Material [file supp_29_5_495__index.html]

Supplemental Material 

# The DREAM complex promotes gene body H2A.Z for target repression

## Supplemental Material

**Files in this Data Supplement:**

- Supplemental Material.pdf
- Supplemental TableS2.pdf
- Supplemental TableS1.xlsx
- Supplemental TableS3.xlsx
- Supplemental TableS54.xlsx
- Supplemental TableS5.xlsx
- Supplemental Table S6.xlsx
- Supplemental Table S7.xlsx
- Supplemental TableS8.xlsx
- Supplemental TableS9.xlsx
