# Supplementary material for: The DREAM complex promotes gene body H2A.Z for target repression
Source: Genes Dev. 2015 Mar 1;29(5):495–500. doi: 10.1101/gad.255810.114 (PMC4358402; doi:10.1101/gad.255810.114)
Supplement: Supplemental Material [file supp_29.5.495_Supplemental_Material.pdf]

Supplemental material for Latorre et al

Supplemental methods

Data processing and plotting

ChIP-seq and RNA-seq reads were aligned to the WS220/ce10 assembly of the *C. elegans* genome using BWA v. 0.6.2 (Li and Durbin 2009) with default settings (BWA-backtrack algorithm). The SAMtools v. 0.1.18 'view' utility was used to convert the alignments to BAM format.

Aligned ChIP-seq reads were extended to the expected insert size (200bp) and reads with low BWA mapping quality (<10) were filtered out to create primary coverage tracks. ChIP-seq data were normalized using the BEADS algorithm (Cheung et al. 2011) implemented in R; BEADS scores are linear enrichment values relative to input. For analyses, BEADS scores were log2 transformed and/or z-scored, as indicated. Where available we used TSSs recently identified based on capped RNA sequencing (Chen et al. 2013; Kruesi et al. 2013). If none were available, we used Wormbase annotated TSSs. Average signal plots and heatmaps for histone marks and DNA associated factors were created using in-house tools. Venn diagrams were generated using the R package VennDiagram (Chen and Boutros 2011). Statistical analyses of Venn overlapping regions were performed using hypergeometric probability functions in R. Motif analyses were performed on L3 DREAM-8 peak overlap regions, using the Seqpos motif tool in the Cistrome toolbox, with default parameters (Liu et al. 2011).

Peak calls

Peak calling for ChIP-seq tracks were done using the wignorm utility from MACS14 (Zhang et al. 2008). Wignorm operates on primary experimental and input coverage tracks and was used with the following settings: 1e-10 p-value

cut-off, 750bp window size and 150bp minimum length of peak. For each experiment, peak calls were done on individual replicates. Peaks were retained only if they were present in all biological replicates.

DREAM-8 regions were defined as those present in all eight L3 or embryo stage DREAM factor peak calls. Peaks that overlapped with High Occupancy Target (HOT) regions (Niu et al. 2011) were excluded from bioinformatic analyses. Overlaps of genes with peak regions were determined using BedTools (v2.20.1; (Quinlan and Hall 2010)). A peak was assigned to a gene if the midpoint of the peak region was located within +/- 500 bp of a Wormbase transcript start site (WS235 gene models lifted over to genome build ce10/WS220). In analyses of DREAM-associated genes, genes were excluded if they were associated with a HOT region or were downstream genes in operons.

RNA-seq expression analyses

An exon model based on WS235 genes was lifted over to ce10/WS220. Tag counts for each gene were extracted from BAM alignment files using HTSeq method working in ”union” mode. These values were used to build the expression matrix. Differential gene expression between N2, *lin-35* mutant, and *htz-1* mutant was tested using DESeq2 (Love et al. 2014). Supplemental Table S5 gives log2 FC, statistical significance estimates, and RPKM values for each gene. Cut-offs for differential expression were DESeq2 FDR < 0.001, Fold change > 1.5 or < -1.5, and RPKM > 1. To avoid small differences in developmental stages from contributing to apparent gene expression differences, we also excluded genes whose wild-type expression oscillates repeatedly during larval development. These were identified as follows. Using a reference gene expression time series (Kim et al. 2013), RPKM RNA-seq values were transformed using the ‘arcsinh’ function in R and quantile normalized using the ‘NormalizeBetweenArrays’ function with option ‘quantile’ in the ‘limma’ package. Independent component analysis (ICA) was performed using the ‘fastICA’ package, extracting twelve

independent components. We used the two most strongly oscillatory components to identify oscillating genes (Supplemental Table S7). Direct DREAM targets were defined as genes bound by DREAM-8 in L3 and upregulated in L3 *lin-35* mutants. Indirect DREAM targets were defined as genes not bound by any DREAM member in L3 but upregulated in L3 *lin-35* mutants. Unregulated DREAM-bound genes were defined as genes bound by DREAM-8 in L3 but not misregulated in L3 *lin-35* mutants (DESeq2 FDR range: $0.5 < x < 1$).

Analysis of HTZ-1 signal on gene bodies

The average HTZ-1 ChIP-seq signal on gene body regions (from 500bp downstream of the Wormbase gene start to the gene end, excluding genes under 500bp in length) was extracted from BEADS normalized tracks using the bigWigSummary utility from UCSC user tools (Kent et al. 2010). These values were used to define genes having the top 5% of gene body HTZ-1 signal and to perform tests for differential gene body signal between wild-type (N2) and *lin-35* mutant background replicates using DeSeq2. Mean signals, log2 fold change (FC) between wild-type and mutant samples, and the statistical significance estimate (p-value) of this change are given in Supplemental Table S6.

Supplemental Figures and legends

Latorre *et al*., Figure S1

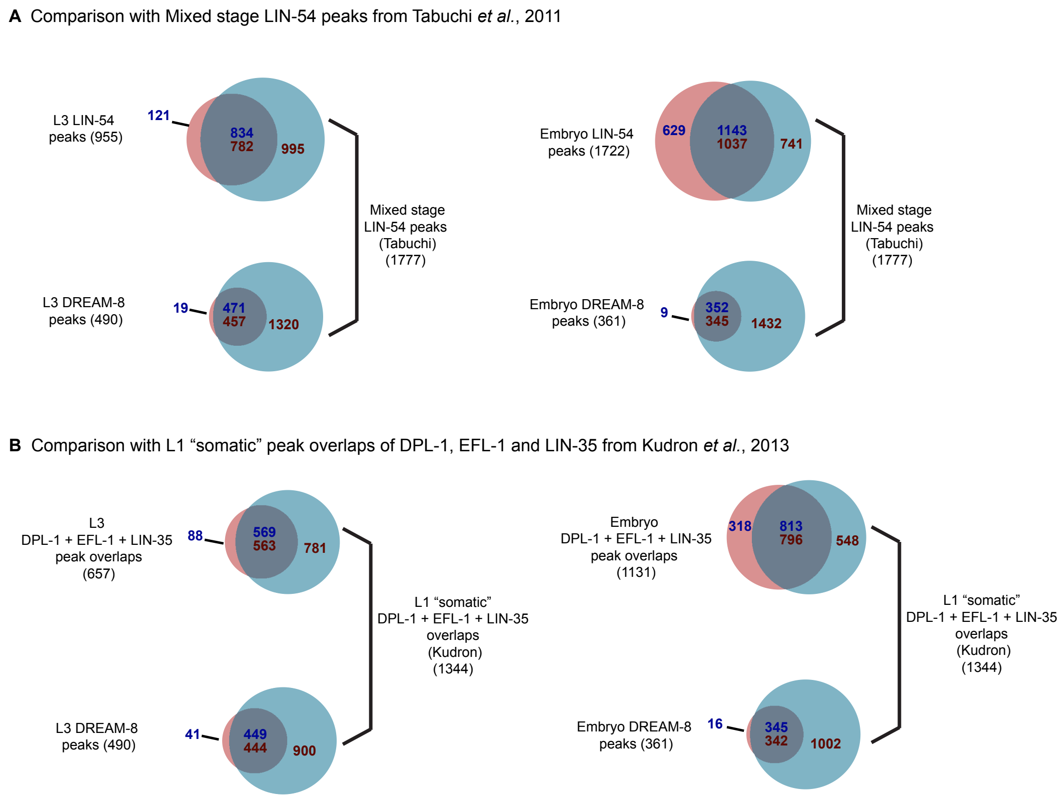

Supplemental Figure S1: Comparison of DREAM peaks with published datasets. Venn diagrams of intersecting genomic regions between L3 and Embryo DREAM subunit peaks, DREAM-8 peaks, and previously published DREAM member datasets. (A) Intersections with mixed stage LIN-54 peaks from Tabuchi *et al*., 2011. (B) Intersections with L1 stage "somatic" peak overlaps from Kudron *et al*., 2013.

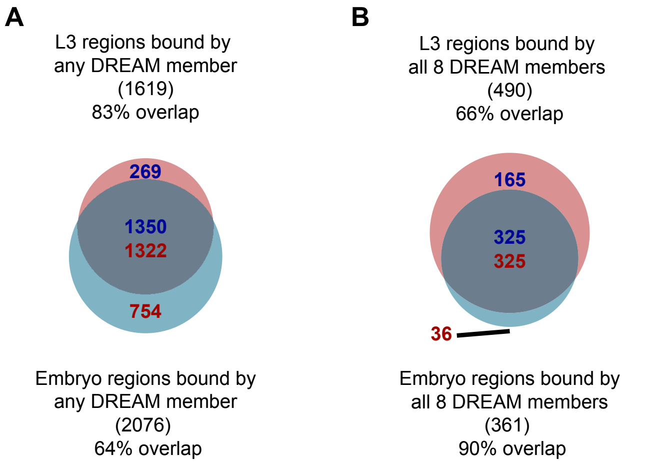

Supplemental Figure S2: Comparison of DREAM peaks between L3 and Embryo stages. (A) Venn diagram of regions bound by 1 or more DREAM member proteins in L3 and Embryo stages. (B) Venn diagram of regions bound by all 8 DREAM member proteins (DREAM-8) in L3 and Embryo stages.

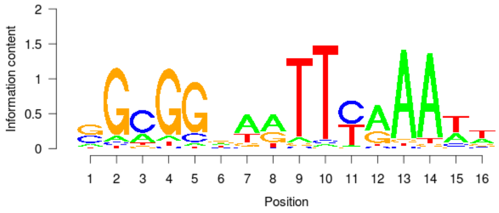

Motif 1
hits: 303
zscore: -26.444

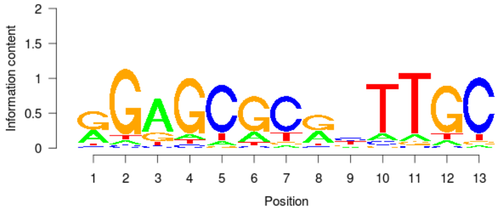

Motif 2
hits: 286
zscore: -24.536

Supplemental Figure S3: Motifs associated with DREAM-8 genes. The two most significant extended consensus motifs (> 10 bp) derived from Cistrome SeqPos motif analysis (Liu et al., 2011), for the 490 regions bound by all 8 DREAM member proteins in L3s. Motif 1 is closely related to the previously identified LIN-54-bound Motif 1 (see Fig. 3E in Tabuchi *et al*., 2011). Motif 2 is closely related to a previously identified “somatic E2F” motif (see Supplemental Fig. 7 in Kudron *et al*. 2013).

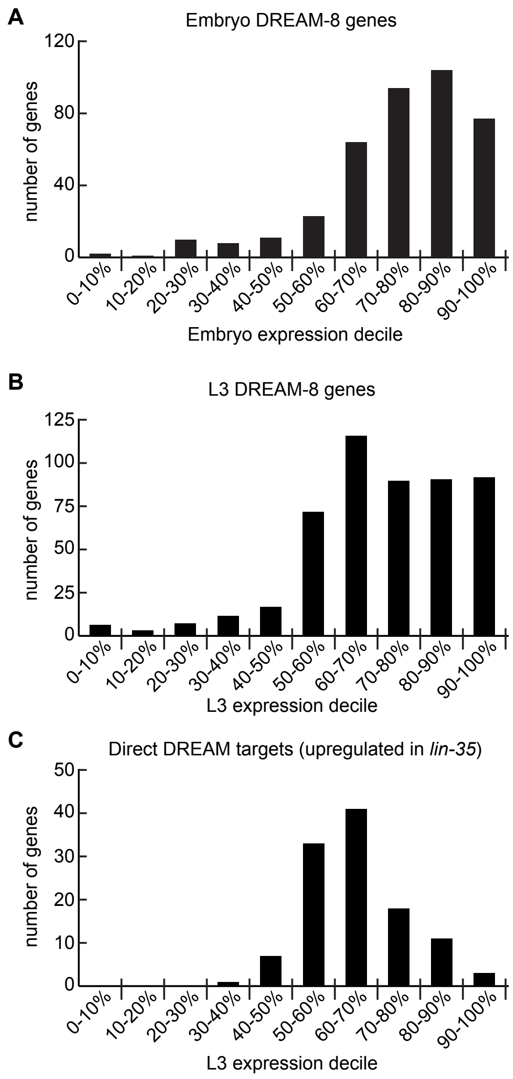

Supplemental Figure S4: Expression deciles of DREAM-bound genes and DREAM-bound genes upregulated in *lin-35* mutants, based on expression data from Hillier *et al*., 2009. (A) Embryo DREAM-8 genes. (B) L3 DREAM-8 genes. (C) Direct DREAM targets: genes bound by DREAM-8 in L3 and upregulated in *lin-35* mutant L3.

Latorre et al; Supplemental Figure S5

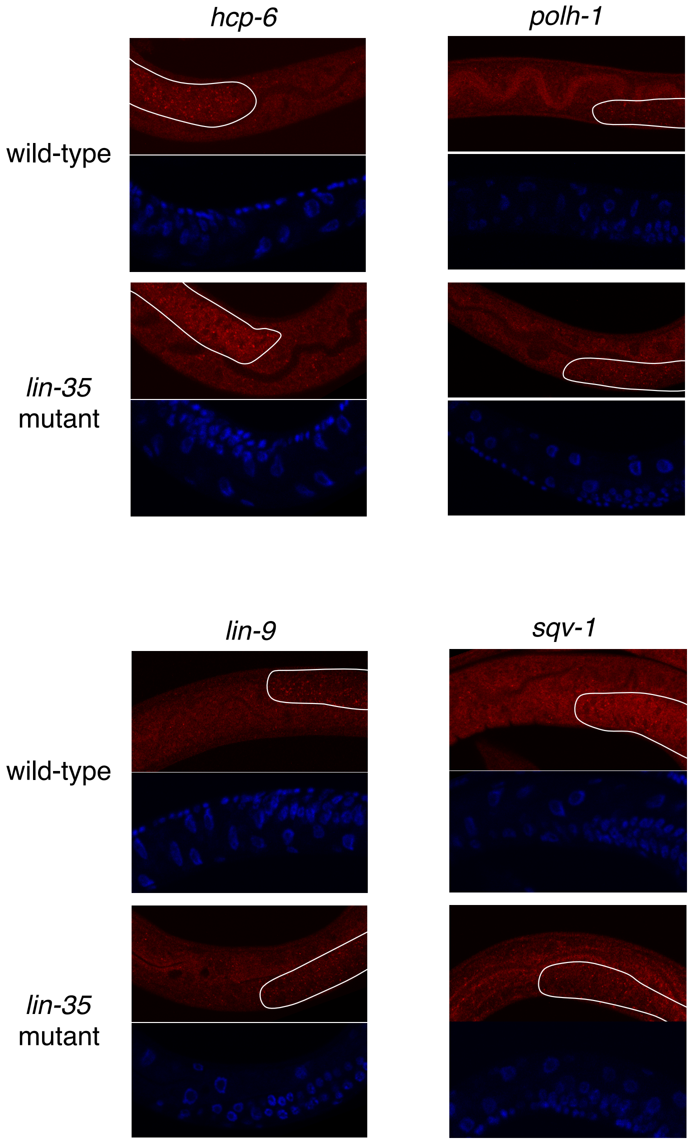

Supplemental Figure S5: Single molecule RNA FISH analysis of direct DREAM targets *hcp-6, polh-1,* and *lin-9* and non-target *sqv-1* in wild-type and *lin-35* L3 larvae. Top: RNA FISH probe signal, Bottom: DAPI. The germ line is outlined in white. There are more RNA FISH foci in both soma and germ line of *lin-35* compared to wild-type.

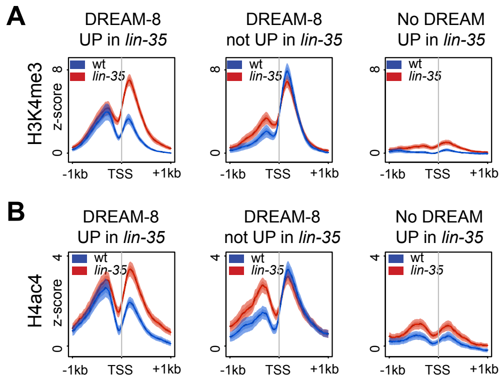

Supplemental Figure S6: Patterns of H3K4me3 and H4ac4 on DREAM-8 genes. Average z-score profiles of normalized signal/input for indicated features and gene sets at the L3 stage (blue, wild-type; red, *lin-35* mutants). (A) H3K4me3 +/- 1kb anchored at the TSS. (B) H4ac4 (H4 acetylated at K5, K8, K12, and K16) +/- 1kb anchored at the TSS.

Supplemental Table Legends:

Supplemental Table S1: (.xlsx file) Genomic locations of DREAM peaks

Worksheet 1: Locations of L3 8 DREAM peak overlaps

Worksheet 2: Locations of L3 8 DREAM peak overlaps excluding those overlapping regions with HOT values ≥ 15

Worksheet 3: Locations of Embryo 8 DREAM peak overlaps

Worksheet 4: Locations of Embryo 8 DREAM peak overlaps excluding those overlapping regions with HOT values ≥ 15

Worksheet 5: Locations of peak overlaps between replicates for individual DREAM member proteins (L3)

Worksheet 6: Locations of peak overlaps between replicates for individual DREAM member proteins (Embryo)

Supplemental Table S2: (PDF file) Summary of regions bound by DREAM members at L3 and Embryo stage

Supplemental Table S3: (.xlsx file) DREAM peaks categorized by genomic features.

Worksheet 1: Promoter-associated DREAM peaks (L3 stage)

Worksheet 2: Gene-body-associated DREAM peaks (L3 stage)

Worksheet 3: Intragenic DREAM peaks (L3 stage)

Worksheet 4: List of all genes with DREAM-bound promoters (L3 stage)

Worksheet 5: Promoter-associated DREAM peaks (L3 stage)

Worksheet 6: Gene-body-associated DREAM peaks (L3 stage)

Worksheet 7: Intragenic DREAM peaks (L3 stage)

Worksheet 8: List of all genes with DREAM-bound promoters (L3 stage)

Supplemental Table S4: (.xlsx file) List of genes misexpressed in *lin-35* and *htz-1* mutants

Worksheet 1: Genes upregulated in *lin-35* vs. wild-type

Worksheet 2: Genes downregulated in *lin-35* vs. wild-type

Worksheet 3: Genes upregulated in *htz-1* vs. wild-type

Worksheet 4: Genes downregulated in *htz-1* vs. wild-type

Worksheet 5: All genes

Supplemental Table S5: (.xlsx file) Gene Ontology analysis

Worksheet 1: *lin-35* upregulated direct DREAM target genes (L3 stage)

Worksheet 2: L3 stage DREAM bound genes

Worksheet 3: Embryo stage DREAM bound genes

Supplemental Table S6: (.xlsx file) Analysis of gene body HTZ-1 levels in wild-type and *lin-35*

Supplemental Table S7: (xlsx file) Genes whose wild-type expression oscillates repeatedly during larval development.

Supplemental Table S8: (xlsx file) Antibody information

Supplemental Table S9: (xlsx file) Locations of data files in GEO and modmine

References

Chen H, Boutros PC. 2011. VennDiagram: a package for the generation of highly-customizable Venn and Euler diagrams in R. BMC bioinformatics **12**: 35.

Chen RA, Down TA, Stempor P, Chen QB, Egelhofer TA, Hillier LW, Jeffers TE, Ahringer J. 2013. The landscape of RNA polymerase II transcription initiation in C. elegans reveals promoter and enhancer architectures. Genome research **23**: 1339-1347.

Cheung MS, Down TA, Latorre I, Ahringer J. 2011. Systematic bias in high-throughput sequencing data and its correction by BEADS. Nucleic acids research **39**: e103.

Hillier LW, Reinke V, Green P, Hirst M, Marra MA, Waterston RH. 2009. Massively parallel sequencing of the polyadenylated transcriptome of C. elegans. *Genome research* **19**(4): 657-666.

Kent WJ, Zweig AS, Barber G, Hinrichs AS, Karolchik D. 2010. BigWig and BigBed: enabling browsing of large distributed datasets. Bioinformatics (Oxford, England) **26**: 2204-2207.

Kim D, Grun D, van Oudenaarden A. 2013. Dampening of expression oscillations by synchronous regulation of a microRNA and its target. Nature genetics **45**: 1337-1344.

Kruesi WS, Core LJ, Waters CT, Lis JT, Meyer BJ. 2013. Condensin controls recruitment of RNA polymerase II to achieve nematode X-chromosome dosage compensation. eLife **2**: e00808.

Kudron M, Niu W, Lu Z, Wang G, Gerstein M, Snyder M, Reinke V. 2013. Tissue-specific direct targets of Caenorhabditis elegans Rb/E2F dictate distinct somatic and germline programs. *Genome biology* **14**(1): R5.

Li H, Durbin R. 2009. Fast and accurate short read alignment with Burrows-Wheeler transform. Bioinformatics (Oxford, England) **25**: 1754-1760.

Liu T, Ortiz JA, Taing L, Meyer CA, Lee B, Zhang Y, Shin H, Wong SS, Ma J, Lei Y et al. 2011. Cistrome: an integrative platform for transcriptional regulation studies. Genome biology **12**: R83.

Love MI, Huber W, Anders S. 2014. Moderated estimation of fold change and dispersion for RNA-seq data with DESeq2. Genome biology **15**: 550.

Niu W, Lu ZJ, Zhong M, Sarov M, Murray JI, Brdlik CM, Janette J, Chen C, Alves P, Preston E et al. 2011. Diverse transcription factor binding features revealed by genome-wide ChIP-seq in C. elegans. Genome research **21**: 245-254.

Quinlan AR, Hall IM. 2010. BEDTools: a flexible suite of utilities for comparing genomic features. Bioinformatics (Oxford, England) **26**: 841-842.

Tabuchi TM, Rechtsteiner A, Strome S, Hagstrom KA. 2014. Opposing activities of DRM and MES-4 tune gene expression and X-chromosome repression in Caenorhabditis elegans germ cells. *G3* **4**(1): 143-153.

Zhang Y, Liu T, Meyer CA, Eeckhoute J, Johnson DS, Bernstein BE, Nusbaum C, Myers RM, Brown M, Li W et al. 2008. Model-based analysis of ChIP-Seq (MACS). Genome biology **9**: R137.
