# Supplementary material for: The DREAM complex promotes gene body H2A.Z for target repression
Source: Genes Dev. 2015 Mar 1;29(5):495–500. doi: 10.1101/gad.255810.114 (PMC4358402; doi:10.1101/gad.255810.114)
Supplement: Supplemental Material [file supp_29.5.495_Supplemental_TableS2.pdf]

A

DREAM member	L3 stage regions bound	Embryo stage regions bound
LIN-35	676	1234
DPL-1	1002	1559
EFL-1	1133	1480
LIN-9	1078	1614
LIN-37	771	1413
LIN-52	533	1599
LIN-53	896	407
LIN-54	955	1722
Regions bound by any DREAM member	1619	2076
Regions bound by all 8 DREAM members	490	361

B

Number of DREAM members bound	L3 regions with n DREAM members bound	Embryo regions with n DREAM members bound
1	486	340
2	173	174
3	100	115
4	101	111
5	82	95
6	85	162
7	81	723
8	484	359
